# Effect of Thermal Processing on Physicochemical and Antioxidant Properties of Raw and Cooked *Moringa oleifera Lam*. Pods

**DOI:** 10.1155/2022/1502857

**Published:** 2022-11-16

**Authors:** Abdur Razzak, Keya Rani Roy, Ummay Sadia, Wahidu Zzaman

**Affiliations:** ^1^Bangladesh Institute of Research and Training on Applied Nutrition (BIRTAN) Regional Office, Sunamganj 3000, Bangladesh; ^2^Department of Food Engineering and Tea Technology, Shahjalal University of Science and Technology, Sylhet 3114, Bangladesh; ^3^Department of Botany, University of Dhaka, 1200, Bangladesh

## Abstract

Moringa is regarded as a miracle tree because all components of the plant, including the roots, leaves, pod, and flowers, have significant nutritional and therapeutic value. *Moringa oleifera Lam*. pods have excellent antioxidant characteristics and are a good source of protein, carbohydrate, fat, vitamins, beta-carotene, amino acids, and phenolic compounds. The pods of *Moringa oleifera Lam*. were collected from the local market of Sunamganj, and their nutritional value was assessed in raw condition and after thermal processing. The goal of this research was to observe how the thermal temperature affected the antioxidant and physicochemical qualities of thermally-processed *Moringa oleifera Lam*. pods. Thermal treatment diminished the amount of crude protein, fat, carbohydrate, ash, ascorbic acid, and beta-carotene in the pods, as well as DPPH, total phenol content, and total flavonoid content. The moisture percentage of raw and thermally-processed *Moringa oleifera Lam*. pods was determined to be 83.12%, 86.03% with a total ash level of 2.01%, and 1.8%, respectively. The crude protein, fat content, and carbohydrate were 3.0%, 0.1%, and 3.2%, respectively, in thermally-processed pods. The values for total phenol content, total flavonoid content, vitamin C, DPPH free radical scavenging activity, and *β*-carotene were 28.13 mg, 2.98 mg, 38.23%, 3.98 mg, and 0.12 mg, respectively, in raw samples whereas 24.56 mg, 2.72 mg, 3.50 mg, 34.32%, and 0.0904 mg, respectively, in thermally-processed samples. According to the findings, *Moringa oleifera Lam*. pods have high nutritional content and thus can be used as an excellent source of diet, and even after thermal processing, a significant nutritive value remains in the *Moringa oleifera Lam*. pods.

## 1. Introduction

*Moringa oleifera* belongs to the family *Moringaceae*, which is predominantly found in the northwestern, Indian subcontinent, and Asia, according to Abdel-Latif et al. [1]. The remarkable moringa tree grows swiftly and can reach heights of 5 to 12 meters [2]. Moringa is drought resistant and can grow with very little water or simply with rainwater entering its tuberous root, according to Dzuvor et al. [3] and Raja et al. [4]. It can thrive in a wide variety of soils, including impoverished soils, though it favors neutral or slightly acidic pH. All of the components of the moringa plant, including the stem, pod, fruit, flowers, and shells, are edible and useful for a number of purposes, such as disease resistance, sanitation, nutrition, industrial applications, and environmental protection. They also contain corresponding amounts of irons, vitamin C, proteins, and calcium ([1, 5].

Leaves, seeds, and the majority of the plant's components fruit or pods, roots, stems, and bark are all used as medications or foodstuffs in different regions of the world; the leaves are a decent supply of macro- and micronutrients, like protein and plenty of vitamins [6]. *Moringa oleifera* has a long history of therapeutic usage in ayurvedic medicine and other complementary and alternative medicine systems, according to Jawonisi et al. [7]. Nitrile, mustard oil glycosides, and thiocarbamate glycosides, which are found in moringa leaves, have shown to reduce blood pressure. Moringa plant alkaloid closely resembles ephedrine in action and can be used to treat asthma, according to a long-standing report. The tree's seed pods are used as vegetables and have a flavor like asparagus, according to Shareef et al. [8].

Additionally, the leaves of *M. oleifera* are a fantastic source of phytonutrients like tocopherols and carotenoids. When combined with a healthy diet, these supplements are known to scavenge free radicals and may have immunosuppressive effects [9]. Moringa leaves are used to alleviate malnourished people and because of their low calorific value, they are also used to treat obese people; their pods are high in fiber, which helps to prevent colon cancer and ease digestive problems, according to Gopalakrishnan et al. [10].

Moringa pods (fruits) are one of the most nutritive, rich in proteins and amino acids necessary for health and proper physical function, as well as calcium and potassium, and useful for promoting good health and alleviating a variety of ailments due to a variety of vitamins and micronutrients ([11, 12]. Oral administration of a hydroalcoholic extract of *M. oleifera* pod was found to increase liver enzymes involved in Phase I and Phase II reactions, such as cytochrome b5, cytochrome P450, catalase, glutathione-peroxidase, glutathione reductase, and glutathione S-transferees, which are responsible for xenobiotic substance detoxification and inhibit skin papillogenesis, according to Promkum et al. [13].

Moringa pod is a widely consumed vegetable in Bangladesh. The majority of *Moringa oleifera* research focuses on the leaves and seed, with only a few studies focusing on the pods; like this study focused on the effect of different cooking methods on moringa pods [14], moringa pod's fortified products [12], and turbidity removal from surface water [15]. However, no literature has been found that compares the nutritional value between thermally-processed and raw *Moringa oleifera Lam*. Thus, the current research primarily focused on the effect of thermal processing on *Moringa oleifera Lam*. pod's physicochemical and antioxidants properties.

## 2. Materials and Methods

### 2.1. Samples and Sample Preparation

Fresh moringa (*Moringa* oleifera) pods locally known as “Sajna” were obtained from Sunamganj district of Bangladesh. All pods were washed with distilled water. For the research work, seeds were removed from these pods. Then, the raw pods were divided into two groups. Then, the raw samples were made as paste by a blender. The other raw pods were soaked in a water bath at 100°C for 30 minutes. The pods were blended as a fine paste using an electric blender and kept in an airtight container in a chiller for further analysis; and by this process, two samples were made for analysis. The raw pods used as control samples in the study. Most of the laboratory analyses were performed in the Department of Food Engineering and Tea Technology, Shahjalal University of Science and Technology, Sylhet, Bangladesh.

### 2.2. Moisture Content Determination

The method outlined by Rajput et al. [16] was used to determine the moisture content of the dried moringa pod powder. First, a crucible was filled with the paste, which was then weighed (*W*_1_). It was then dried in an oven dryer for 24 hours at 105°C before being weighed once more (*W*_2_). The following equation was used to determine the sample's moisture content:

(1) $$\begin{array}{c} \text{Moisture content}=\frac{\left(W_{1}-W_{2}\right)}{W}\times 100. \end{array}$$

### 2.3. Ash Content Determination

Using a technique developed by Akubugwo et al. [17], the ash content of raw materials and thermally-processed *Moringa oleifera* pods was determined. About 3 g of fresh and thermally-processed moringa pod paste was taken into a crucible. These samples were then heated at 550°C for 8 hours in a muffle furnace, and the residue was determined.

### 2.4. Fat Content Determination

A technique described by Ravichandran and Parthiban [18] was used to assess the fat content of raw and thermally-processed moringa pod pastes. First, in a separating funnel, 5 g of paste from raw and thermally-processed moringa was combined with 25 ml of a 2 : 1 mixture of methanol and chloroform. After that, 5 ml of sodium chloride at 0.9% was added. The separating funnels revealed three levels. A preweighed beaker was filled with the bottom layer, which was then heated in a water bath at 80°C. The weight of the beaker containing the residue was then calculated. Thus, the fat content was calculated by inserting the value of a beaker.

### 2.5. Protein Content Determination

According to Offor et al. [19], they described the Kjeldahl method, which was used to evaluate the protein content of moringa pod paste. First, a digestion mixture and sulfuric acid were used to digest the paste. Following digestion, the digested sample was subjected to distillation in a distillation chamber. Then, HCl measured the titrate value. This technique was used to determine the nitrogen content %. The nitrogen percentage was multiplied by a conversion factor to get the protein content.

### 2.6. Carbohydrate Content Determination

According to the method developed by Agrawal et al., (2015) the amount of carbohydrates was estimated. First, 0.1 g of moringa pod paste was cooked for three hours in a water bath. In order to neutralize the sample, solid sodium carbonate was added. By adding distilled water, the sample volume was increased to 100 ml, and it was then centrifuged. For a subsequent procedure, a sample of the supernatant was employed. By adding distilled water, a 0.2 ml sample was converted to 1 ml. After that, the sample was mixed with 1 ml of 5% phenol and 5 ml of 96% sulfuric acid, and its absorbance was measured using a spectrophotometer at 490 nm. Utilizing the glucose standard curve, the percentage of carbohydrates was calculated.

### 2.7. Total Phenol Content

In accordance with the procedure outlined by Amorim et al. [20], the total phenol content was calculated. First, an extract solution was made by mixing 0.5 g of moringa pod paste with 80% methanol. A few minutes later, 0.5 ml of the extract was combined with 8.5 ml of distilled water and 0.5 ml of Folin-Ciocalteu. After adding 1 ml of a 35% sodium carbonate solution to the mixture, the absorbance at a wavelength of 765 nm was measured. The gallic acid standard curve was used to calculate the total phenol concentration.

### 2.8. Total Flavonoid Component

TFC was assessed by the Priecina and Karlina technique (2013) using a modified colorimetric test. The 0.25 ml of the extract and 0.75 ml of distilled water were combined in a test tube, and 5% of a 0.15 ml sodium nitrite (NaNO2) solution was then added to the combination. Then, after waiting for five minutes, 0.3 ml of a 10% aluminum chloride (AlCl3) solution and 1 ml of a 1 M sodium hydroxide solution were added. At 510 nm, the mixture's absorbance was determined using spectrophotometry (Shimadzu UV-1800, Tokyo, Japan). A calibration curve (*Y* = 0.0029*x* + 0.0169) for quercetin was established for the flavonoid contents, and the TFC was then expressed as quercetin equivalent (mg QE/100 g).

### 2.9. Determination of DPPH Free Radical Scavenging Activity

According to Adiletta et al. [21], technique was used to measure the DPPH free radical scavenging activity. The extract solution was initially made. Then, 2 ml of the extract sample was added to 2 ml of the DPPH (0.16 mM) methanol solution, which was then left in the dark. A solution without extract was used as a reagent blank, and the mixture's absorbance was calculated at 517 nm wavelength.

(2) $$\begin{array}{c} \%\text{DPPH radical scavenging activity}=\frac{{\text{Abs}}_{\text{control}}-{\text{Abs}}_{\text{sample}}}{{\text{Abs}}_{\text{control}}}. \end{array}$$

### 2.10. Determination of Vitamin C Content

The method described by Ranganna was used to determine the vitamin C concentration (1986). First, a conical flask was filled with 5 ml of normal ascorbic acid and 5 ml of metaphosphoric acid. For the purpose of titrating the sample, dye solution was placed in a burette. Through this technique, dye factor was produced. After that, a 5 g sample was titrated by adding 3% HPO 3.

### 2.11. Determination of *β*-Carotene

The amount of beta-carotene was measured using a technique reported by Biswas et al. [22]. A test tube was filled with 1 g of sample and 5 g of cold acetone. After centrifuging the mixture, the supernatant was transferred to a different test tube. The absorbance of the extract and standard-carotene solution was measured using a spectrophotometer at 421 nm wavelength after creating the standard-carotene content.

### 2.12. Statistical Analysis

Using the renowned program Minitab (Version 19.2020.1), the findings were presented as the mean standard deviation (SD). Tukey's test and analysis of variance (ANOVA) were used to compare means. The least significant difference was calculated for significant data at *P* < 0.05.

## 3. Results and Discussion

The moisture content of *Moringa oleifera* raw and thermally-processed pods was found to be 83.12% and 86.03%, respectively, in Table 1. For the thermal effect in water bath, pod samples gained 3.38% moisture content.

The higher moisture level in the thermally-processed moringa pods suggests that thermally-processed *Moringa oleifera* pods may become more sensitive to microbial growth. This is to be expected, given that the material was preserved to maintain its moisture content. The overall ash content was discovered to be reduced from 2.01% to 1.8% in raw and thermally-processed pods, respectively. The ash level of foods is widely accepted as a quality indicator for evaluating their functional characteristics. The ash content of organic food is commonly used to determine the mineral content. When proximate analyses of thermally-processed samples have been compared to raw pod sample findings, all studies, except for moisture content, showed a decline in thermally-processed pods. The findings were almost identical to those of Shareef et al. [8]. Thermal processing resulted in a significant reduction in crude protein, reducing proteins from 3.32% to 3.0%, and overall 9.64% crude protein was reduced. Thermal temperature significantly reduced the fat content; it causes 16.67% loss of fat compared to raw samples. The amount of protein found in *Moringa oleifera* pods suggests that the plant might be consumed by humans. In *Moringa oleifera* pods, the carbohydrate content was determined to be 4.3% and 3.2% in the raw and cooked samples, respectively, which caused 25.58% lost in the carbohydrate content of thermal samples. *Moringa oleifera* pods were found to be a good source of energy in this study. These declines could have occurred as a result of the heat. Thermal processed samples, on the other hand, absorb higher moisture than raw samples as a result of water absorption. The results, which were determined using a wet weight basis, were discovered to be identical to those reported by Shareef et al. [8], who calculated the proximate analysis of drumstick pods.

Plants are rich in phenolic antioxidants, which are beneficial to the body as well as serving as a suppressor of free radicals. Their ability to neutralize free radicals is impressive to hydroxyl groups, according to Ghafar et al. [23]. TPC in the extract of *M. oleifera Lam*. 28.13 and 24.56 mg was found in raw and thermally-treated sample pods, respectively, as shown in Table 2. TPC was lower in the thermal process samples than in the fresh samples, with a TPC value of 32.07 mg according to Abdulkadir et al. [24]. This could be due to the samples being exposed to high air temperatures. This could cause the phenolic chemicals to degrade too quickly in the heat. On the other hand, it is possible that this is the effect of the extended wait. Cooking time led to longer oxygen exposure, and is harmful, is favorable for oxidase enzymes to carry out the oxidation procedure. In case of TPC, overall 12.69% losses occurred.

Fruits and vegetables, as well as spices, are significant components of the human diet, in part because they comprise natural antioxidants, primarily flavonoids that may help maintain normal physiological activities. Raw and thermally-treated pod samples have 2.98 mg and 2.72 mg TFC content, respectively. Heat causes the hydrolysis of glycosides and the release of flavonoid monomers [25]. During the boiling process, which is why the flavonoid content has reduced 8.73% compared to the fresh sample containing 2.98 according to this paper [24].

Based on the mechanism by which antioxidants restrict lipid oxidation, resulting in DPPH radical scavenging and evaluating free radical scavenging capability, the DPPH assay is used to predict antioxidant activity. The raw samples have 38.23%, and cooked samples have 34.32% DPPH free radical scavenging activity compared to the fresh samples. DPPH was found on this paper [24]. Thermal treatment causes 10.23% reduction in DPPH free radical scavenging activity.

Vitamin C is an essential dietary component for human health. Vitamin C should be kept saturated in the human body to preserve excellent health and to prevent colds. But because of how delicate it is, it is readily damaged by a variety of things, most notably light and heat. According to Ahmed et al. [26], raw *Moringa oleifera* pods have 3.98 mg of vitamin C, which was comparable, and fresh *M. oleifera* pods have vitamin C content ranging from 3.96 mg to 8.27 mg. Due to its heat sensitivity and consequent reduction with rising temperature, the thermally-treated sample had 3.50 mg of vitamin, according to Raja et al. [4]. Vitamin C was therefore decreased by 12.06% due to the heat effect. In thermal samples, beta-carotene content was 0.12 mg and 0.0904 mg in raw and processed samples, respectively. Fresh pods had 0.11 mg beta-carotene found on paper [28]. The findings suggest that cooking reduces beta-carotene levels significantly. In this study, cooking found the reduction of 24.67% beta-carotene. When vegetables are placed in boiling water and cooked for the longer time, beta-carotene losses are claimed to be increased.

## 4. Conclusions

The research shows that the *Moringa oleifera Lam*. pods have better physiochemical and antioxidant capabilities that greatly benefit our diet. The raw pods have higher nutritional value than thermally-treated samples, although thermal treatment could not reduce a large amount of nutrients. Because several components of pods are heat sensitive, the traditional methods of thermal processing for eating them as vegetables reduce their nutritive value. However, because a trivial amount of nutrient content is diminished, providing pods as a vegetable relish that could significantly provide nutritional benefits to the individuals. Due to time and financial constraints, we were unable to compare the nutritional value of various variety *Moringa oleifera* pod samples and unable to create new products exploiting their nutritional value.

## Figures and Tables

**Table 1:** Effect of thermal processing on the physiochemical properties of *Moringa oleifera* pods.

Serial no.	Chemical characteristics	Raw pods results (percentage)	Cooked pods results (percentage)	Percentage losses after cooking
1	Moisture	83.12 ± 1.8^*a*^	86.03 ± 2.05^*b*^	3.38%
2	Ash	2.01 ± .20^*a*^	1.8 ± .33^*a*^	10.45%
3	Protein	3.32 ± .12^*a*^	3.0 ± .20^*b*^	9.64%
4	Fat	12 ± .07^*a*^	0.1 ± .08^*b*^	16.67%
5	Carbohydrate	4.3 ± .18^*a*^	3.2 ± .12^*a*^	25.58%

Samples in the same raw with different superscript letters differ significantly at *P* < 0.05.

**Table 2:** Effect of thermal processing on the antioxidant properties of *Moringa oleifera* pods.

Serial no	Content	Raw pods	Thermally-processed pods	Percentage losses after thermal process
1.	Total phenol content (mg/100 g)	28.13 ± 2.3^*a*^	24.56 ± 1.2^*b*^	12.69%
2.	Total flavonoid content (mg/100 g)	2.98 ± .12^*a*^	2.72 ± .20^*a*^	8.73%
3.	DPPH (%)	38.23 ± 2.78^*a*^	34.32 ± 1.8^*b*^	10.23%
4.	Vitamin C (mg/100 g)	3.98 ± .22^*a*^	3.50 ± .15^*b*^	12.06%
5.	*β*-carotene (mg/100 g)	.12 ± .003^*a*^	0.0904 ± .001^*a*^	24.67%

Samples in the same raw with different superscript letters differ significantly at *P* < 0.05.

## Data Availability

The study data are preserved in our laboratory. It can be provided if needed.
